# Chronic Nasal Catarrh

**Published:** 1876-12

**Authors:** E. C. Guhrung

**Affiliations:** St. Louis, late of Denver, Colorado


Chronic Nasal Catarrh.—*By E. C. Guhrung, M.D., St. Louis, late of Denver, Colorado.*—The almost universal prevalence of this disease invests it with general interest. May this serve as my apology for touching once more this frequent field.

I do not intend to exhume the literature of the past on this subject, nor write an exhaustive treatise on its pathology, as this can be found in the text-books. My intention is merely to report the results of my observations and to explain my treatment, which for several years has been followed with satisfactory results.

It is the uncomplicated chronic catarrh of the posterior nares that forms the subject of this paper; uncomplicated by any diathesis or specific disease whatever, not rendered incurable by previous heroic (?) treatment, nor dependent on polypus or tumors of any kind.

The dry state of the atmosphere of Colorado appears to be favorable to its development to an extent relatively greater than in countries differently situated. The moisture of the nasal mucous membrane will be rapidly exhausted; and, as a natural consequence, the protecting epithelium will become dry, peel off, and act as an additional irritant.

Like other catarrhal diseases, nasal catarrh recognizes multiple causes; *dryness*, which appears to occupy the first rank, as a direct cause; *dampness*, which acts indirectly, by way of a series of acute attacks, from suppression of the cutaneous functions; and mechanical, chemical, and *imponderable irritants*.

It is to its favorite locality on the pharyngeal surface of the soft palate that I wish to draw the attention, as this furnishes the indications for the treatment.

Why the afore-mentioned locality should be the one preferred must be answered theoretically. The air inspired through the nose passes in a straight line to that point, and there undergoing deflection, almost at right angles from its course, must certainly cause the comparatively greatest amount of friction, dessication, and the deposit of the larger quantity of foreign substances it may contain. These causes act uninterruptedly. The mucous membrane will soon be abraided, which abrasions scab over, and finally thick, ropy mucus and muco-purulent masses are formed, which will flow down along the posterior pharyngeal wall to the larynx. The scabs, as well as those masses, with the simultaneous dryness of the pharynx and soft palate, cause the difficulty in swallowing so generally complained of by the patients, and irritate the pharynx and larynx, producing hoarseness and coughing attacks, with an expectoration sometimes simulating that of consumption. Likewise does this tickling of the soft palate and pharynx, and the attempt to get rid of the crusts, etc., cause the subject of this trouble to vomit his meals, in consequence of which a state of inanition is brought about, which may be mistaken for consumption.

*Treatment.*—The disease occupies, as before stated, principally the pharyngeal surface of the soft palate. The indications are to find the means of cleansing the surface referred to from incrustations and mucus, and a method to get medicine in contact with the freshly cleared surface. Among the remedies and modes of application of the same in vogue, there are none, to my knowledge, meeting the above indications satisfactorily. By the nasal douche, if used according to directions, the fluids circulate on the floor of the nose, while at the same time the patient is taught to apply the soft palate to the posterior pharyngeal wall, and the diseased surface is thereby completely protected from the contact of medicated fluids. Snuffiing or throwing fluids or powders into the nose will meet the same fate. Injections from the posterior nares meet with the objection, that the pipe of the syringe effectually protects the affected part. Application of the solid caustic cannot meet with success if the crusts are not primarily removed, and because these applications are too severe, and can therefore not be used sufficiently often. Another objection against all these means is, that they are not altogether free from danger, are painful and entirely too severe. A very simple, and according to my experience, very effectual plan remains:

The patient is directed to lean back his head. With a small syringe, teaspoon, or any other contrivance, let a small quantity of tepid salt water (ʒi–Oi) or bicarbonate of potash in water (ʒi–Oi) flow gently into one of the anterior nares, and direct him to expire gently through the nose with the mouth closed, when some of the fluid will flow down along the pharynx, which fluids he may eject by the mouth, and, leaning forward, empty his nose at the same time. After repeating this process a few times by each nostril, both crusts and mucus will be completely removed. This treatment alone, repeated as often as any crusts can be washed away (first twice, then once a day, then every second day, etc.), will generally cure the disease within a few weeks. The vomiting and hoarseness will disappear likewise. The process of healing may be hastened by using—in the same way as the salt water, and after it—small quantities of weak medicated solutions.

A permanent cure cannot often be expected, as the causes are constantly in operation. Consequently it will be necessary to instruct the patient to recur to the same treatment from time to time as necessity may demand.

Simple chronic catarrh of the anterior nares, with the formation of scabs or crusts, can be cured by letting the crusts alone, and by applying every night on going to bed a lump of lard as high up as can be reached by the little finger in the affected nostril. These crusts can be blown away, without difficulty, in the morning, and soon no more will form. The trouble is then cured until, in consequence of the repetition of its causes, it may recur; when the same application will have the same result.

The principle of this treatment, therefore, consists in: Removal of obstructions and irritants, and substitution of moisture and mild stimulation.

The above method is also applicable for the administration of mild gargles, as they are brought more effectually in contact with the pharynx, and sometimes the larynx, than by most other methods.

If I have succeeded in showing that the rebellious character of this disease to "energetic treatment" may be conquered by removing the immediate cause and mild topical applications, I shall consider myself amply rewarded.—*St. Louis Clin. Record.*

